# Comprehensive evaluation and performance analysis of machine learning in heart disease prediction

**DOI:** 10.1038/s41598-024-58489-7

**Published:** 2024-04-03

**Authors:** Halah A. Al-Alshaikh, Prabu P, Ramesh Chandra Poonia, Abdul Khader Jilani Saudagar, Manoj Yadav, Hatoon S. AlSagri, Abeer A. AlSanad

**Affiliations:** 1 Information Systems Department, College of Computer and Information Sciences, Imam Mohammad Ibn Saud Islamic University (IMSIU), 11432 Riyadh, Saudi Arabia; 2 Department of Computer Science, CHRIST University, Bangalore, 560029 India; 3 Department of Computer Science and Engineering, Guru Jambheshwar University of Science and Technology, Hisar, India

**Keywords:** Heart disease, Prediction, Healthcare, Machine learning, Diseases, Health care

## Abstract

Heart disease is a leading cause of mortality on a global scale. Accurately predicting cardiovascular disease poses a significant challenge within clinical data analysis. The present study introduces a prediction model that utilizes various combinations of information and employs multiple established classification approaches. The proposed technique combines the genetic algorithm (GA) and the recursive feature elimination method (RFEM) to select relevant features, thus enhancing the model’s robustness. Techniques like the under sampling clustering oversampling method (USCOM) address the issue of data imbalance, thereby improving the model’s predictive capabilities. The classification challenge employs a multilayer deep convolutional neural network (MLDCNN), trained using the adaptive elephant herd optimization method (AEHOM). The proposed machine learning-based heart disease prediction method (ML-HDPM) demonstrates outstanding performance across various crucial evaluation parameters, as indicated by its comprehensive assessment. During the training process, the ML-HDPM model exhibits a high level of performance, achieving an accuracy rate of 95.5% and a precision rate of 94.8%. The system’s sensitivity (recall) performs with a high accuracy rate of 96.2%, while the F-score highlights its well-balanced performance, measuring 91.5%. It is worth noting that the specificity of ML-HDPM is recorded at a remarkable 89.7%. The findings underscore the potential of ML-HDPM to transform the prediction of heart disease and aid healthcare practitioners in providing precise diagnoses, exerting a substantial influence on patient care outcomes.

## Introduction

Healthcare encompasses a range of disciplines. It focuses on preventing, diagnosing, treating, and caring for illnesses and ailments that impact people’s and communities’ overall health and welfare^1^. The scope of services is extensive, including diverse offerings such as medical exams, interventions, psychological assistance, and public health activities. Healthcare professionals, encompassing a diverse range of individuals such as physicians, nurses, therapists, and researchers, engage in collaborative efforts to safeguard the whole well-being of patients, surrounding their physical, mental, and emotional health. Alongside clinical treatment, healthcare contains health information, illness prevention, and health advancement, all of which improve the overall quality of life^2^.

Heart disease, an often-seen and frequently consequential medical ailment, has a profound interdependence with the broader healthcare domain^3^. Heart disease is a significant contributor to illness and death worldwide, underscoring the crucial role of healthcare systems in tackling pressing health concerns. Heart disease comprises a range of conditions that impact the heart and blood arteries, including heart failure, coronary artery disease, arrhythmias, and valve abnormalities. Healthcare professionals are crucial in diagnosing heart disease by using medical evaluations, employing modern imaging methods, and conducting diagnostic procedures^4^. The management of the condition involves a comprehensive approach encompassing lifestyle adjustments, pharmaceutical therapies, and surgical procedures, highlighting the need for interdisciplinary cooperation among healthcare professionals. Healthcare efforts that prioritize the promotion of heart-healthy habits, the enhancement of perception, and the advocacy for early detection play a crucial role in mitigating the prevalence of heart disease and enhancing cardiovascular well-being on a broader scope. Examining and controlling cardiovascular disease exemplify the complex interaction between healthcare and the welfare of people and society^5^.

Cardiovascular disorders rank first among the most lethal illnesses. They are well recognized as a significant contributor to mortality on a worldwide scale^6^. Based on statistical data from the World Health Organization, it was reported that cardiac disorders were responsible for about 18 million fatalities in 2016. In the United States, cardiovascular illnesses, including heart disease, hypertension, and stroke, are the leading causes of mortality. Cardiovascular heart disease (CHD) is responsible for about one-seventh of all fatalities in the United States, resulting in an estimated yearly death toll of 3 million individuals^7^. The prevalence of myocardial infarctions in the United States is roughly 7.9 million, accounting for approximately 3% of heart attack incidents among American adults. In the year 2015, a total of 1 million individuals inside a single nation succumbed to fatalities resulting from heart attacks^8^. Based on the findings of a study, the symptoms associated with heart disease include a range of manifestations such as chest tightness, discomfort, pressure, breath shortness, leg shivers, neck pain, abdomen pain, tachycardia, dizziness, bradycardia, fainting, syncope, which skin color modifications, leg swelling, weight loss, and weariness^9^. The symptoms of heart illness, including arrhythmia, myocardia, heart failure, congenital heart disease, mitral regurgitation, and dilated cardiomyopathy, exhibit variation based on their specific nature.

The risk factors associated with heart disease include several aspects such as advanced age, genetic predisposition, tobacco use, physical behaviors, substance misuse, elevated cholesterol levels, hypertension, sedentary lifestyle, obesity, diabetes, psychological stress, and inadequate hygiene practices^10^. The gravity of cardiovascular disease mandates the implementation of a screening protocol for its diagnosis. In the screening procedure, medical professionals provide many diagnostic tests, including blood glucose level testing, cholesterol testing, blood pressure measurement, electrocardiography (ECG), ultrasound imaging, cardiac computer tomography (CT), calcium scoring, and stress testing. The screening procedure requires significant time-consuming manual tasks and human involvement.

The primary contributions of the research are listed below:

- Using the GA and RFEM in advanced feature selection guarantees identifying the most relevant features, reducing interference and augmenting the model’s predictive capability.
- Using feature extraction methodologies extends beyond merely selecting raw attributes, converting the data into a more comprehensive and informative representation. This process enhances the input for later analysis.
- USCOM techniques tackle the prevalent issue of uneven data distribution, enhancing the model’s capacity to generalize well across different classes.
- Using a MLDCNN in classification exploits the ability of deep learning to capture detailed patterns present in complicated databases effectively, improving the model’s prediction performance.
- The AEHOM facilitates the refinement of the model’s parameters, enhancing its performance and promoting effective convergence during training.

The following sections are arranged in the given manner: “Background and literature survey”aims to provide a comprehensive overview of the backdrop around heart disease prediction and thoroughly examine pertinent previous research conducted in this field. “Proposed heart disease prediction system” describes the unique machine learning-based heart disease prediction method (ML-HDPM) approach, which integrates feature selection, feature extraction, UAO, MLDCNN, and AEHOM techniques to achieve precise prediction. “Simulation analysis and findings” presents and analyzes the numerical results, emphasizing the better effectiveness of the ML-HDPM model in predicting heart disease. “Conclusions” provides a concise overview of the obtained results, examines their ramifications, and delineates potential directions for future investigations and improvements within the domain.

## Background and literature survey

Cardiovascular disorders have been widely acknowledged as one of the primary contributors to mortality. Several research investigations have been conducted to examine the use of predictive models in forecasting indicators associated with the likelihood of developing cardiac disorders based on health data found in the existing literature. The latest advancements in machine learning tools and algorithms have stimulated research efforts in creating methodologies and techniques for detecting cardiac disease. Numerous methods have been explored to address this issue, including classification, clustering, etc.

The work conducted by Abdullatif et al. introduces a novel approach that combines supervised infinite feature selection with an enhanced weighted random forest algorithm to detect heart disease^11^. The process is selected to improve the significance of features and the model’s accuracy. The simulation results show significant improvement, achieving an accuracy rate of 92.14%, a sensitivity rate of 92.50%, and a specificity rate of 91.78%. This methodology presents an enhanced approach for detecting cardiac illness, potentially increasing patients’ survival rates.

Muhammad et al. propose a sophisticated computer model to identify cardiac illness precisely^12^. The solution being presented utilizes machine learning methods to get accurate diagnoses. The model demonstrates favorable results, achieving an accuracy rate of 92.42%, a sensitivity rate of 92.40%, and a specificity rate of 92.44%. This work showcases the capacity of intelligent computational models to diagnose cardiac disease with precision and efficiency.

Arnaout et al. provide a novel approach, including using an ensemble of neural networks for the accurate prenatal detection of complicated congenital heart disease^13^. The use of neural networks in this approach enables precise diagnostic outcomes. The ensemble exhibits exceptional performance, attaining an area under the receiver operating characteristic curve value of 0.98. The research emphasizes the possibility of using neural network ensembles to identify fetal cardiac disease.

The study conducted by Alkhodari et al. centers on diagnosing valvular heart disease using convolutional and recurrent neural networks (CNNs and RNNs) applied to phonocardiogram recordings^14^. The selection of CNNs and RNNs is appropriate for data organized in sequences. The findings exhibit a noteworthy outcome, showcasing an overarching precision rate of 95.86%. This underscores the effectiveness of using deep learning techniques in the realm of diagnosing valvular heart disease.

The heart disease diagnosis approach using machine learning classification in the field of e-healthcare is proposed by Li et al.^15^. The research utilizes a range of classification techniques to enhance the precision of identification. The Random Forest classifier has notable performance, with an accuracy rate of 90.47%, a precision value of 0.909, and a recall value of 0.912. This method places significant emphasis on using machine learning techniques within the field of e-healthcare, specifically to accurately identify cases of heart disease.

Dixit et al. demonstrated the use of a cost-effective and portable ECG sensor for the early identification of cardiovascular disease^16^. Their suggested solution utilizes machine learning algorithms to process ECG data. The system encompasses several characteristics, such as capturing, preprocessing, and categorizing ECG signals. The simulation findings demonstrate a high level of accuracy, namely 95.2%, in diagnosing cardiac illnesses. This highlights the potential for using a low-cost ECG sensor as an effective means of early detection.

Alsafi et al. introduced a sophisticated machine-learning system to diagnose coronary heart disease^17^. The presented approach utilizes machine learning methodologies to examine patient data, effectively using their characteristics for precise diagnoses. The simulation results demonstrate a notable level of precision, with an accuracy rate of 97.4%. This highlights their methodology’s potential to improve coronary heart disease diagnosis.

Ali et al. provided a novel healthcare monitoring system to anticipate cardiac disease^18^. The proposed method employs ensemble deep learning and feature fusion techniques to analyze patient data. The technique’s effectiveness is shown by its ability to achieve a prediction accuracy of 92.34% in the context of heart disease, highlighting the promise of ensemble deep learning as a reliable approach for monitoring healthcare.

Gárate-Escamila et al. focused on predicting heart disease using classification models, feature selection techniques, and principal component analysis (PCA)^19^. The suggested methodology emphasizes the importance of feature selection and dimensionality reduction. The simulation findings demonstrate a competitive level of performance, as shown by an accuracy rate of 87.5% obtained via the use of PCA. This outcome highlights the promising capabilities of these approaches in the realm of heart disease prediction.

Sonawane et al. proposed a heart disease prediction model that integrates data and ECG signals using a hybrid clustering approach^20^. The proposed method combines clustering methodologies with machine learning algorithms to achieve precise prediction. The suggested method achieves a classification accuracy of 92.6%, offering the possibility of using hybrid clustering techniques to enhance the prediction of cardiac illness by integrating both traditional data and ECG signals.

The literature review elucidates many methodologies for diagnosing and predicting heart illness, including advanced machine learning, deep learning, and sensor-based data-collecting strategies. Although these methodologies provide encouraging outcomes, several limitations arise, such as inconsistent diagnostic precision and possible issues related to overfitting. The suggested methods effectively tackle these issues by incorporating cutting-edge technology and feature selection procedures, augmenting the accuracy and dependability of heart disease diagnosis and prognosis.

## Proposed heart disease prediction system

The research endeavors to establish a comprehensive framework for achieving precise heart disease prediction, employing a multifaceted approach that integrates advanced machine learning methodologies, feature selection, and dimensionality reduction techniques. By harnessing sophisticated machine learning algorithms, the model adeptly discerns intricate patterns embedded within patient data, utilizing ensemble deep learning and innovative feature fusion strategies. This holistic strategy ensures the accurate and timely prognosis of cardiac disease, furnishing healthcare practitioners with a valuable tool to enhance patient treatment outcomes.

Moreover, the hybrid system encompasses three pivotal stages: data gathering, pre-processing, and classification, each playing pivotal roles in refining the predictive model. During the meticulous pre-processing phase, a series of tasks are meticulously executed to uphold data integrity and model efficacy. These tasks encompass the meticulous imputation of missing data, wherein the ML-HDPM method is proficiently utilized to accurately estimate absent values within the database. Furthermore, exhaustive feature selection endeavors are undertaken to pinpoint the most pertinent attributes for predictive modeling. This process is facilitated by a hybrid technique that synergistically amalgamates the capabilities of genetic algorithm (GA) and recursive feature elimination method (RFEM), facilitating the identification of informative features crucial for precise predictions.

Additionally, to ensure uniformity in the impact of features, a conventional scalar approach is employed to recalibrate the coefficients of all features, aligning their means to 0 and standard deviations to 1, thereby mitigating potential biases stemming from feature scale discrepancies. Within the database, classification is facilitated by delineating between two distinct classes: class 0 and class 1, representing the absence and presence of heart disease, respectively. Specifically, 164 cases are attributed to class 0, indicative of the absence of heart disease, while 139 instances are categorized as class 1, denoting the presence of heart disease. To mitigate the inherent class imbalance, the synthetic minority over-sampling technique (SMOTE) is deployed, ensuring equitable representation of both classes and bolstering the model’s predictive prowess across diverse scenarios.

Implementation of the synthetic minority over-sampling technique (SMOTE) within the research framework entails a systematic approach to redress the inherent class imbalance between class 0 (indicating the absence of heart disease) and class 1 (signifying the presence of heart disease). SMOTE functions by generating synthetic samples from the minority class, thereby augmenting its representation within the dataset. This technique involves selecting individual instances from the minority class and creating synthetic instances along the line segments connecting them to their nearest neighbors. By interpolating between existing instances, SMOTE effectively enhances the diversity of the minority class without introducing biases, thus fostering a balanced representation of both classes. This approach fortifies the model’s predictive capabilities, ensuring robust performance across a myriad of scenarios and enhancing its utility in clinical practice.

Specifically, SMOTE facilitates equitable representation of both classes, enabling the model to learn from a more diverse range of examples and effectively capture the underlying patterns and nuances associated with both the presence and absence of heart disease. As a result, the predictive model becomes more adept at accurately discerning between cases of heart disease and non-heart disease, thereby enhancing its diagnostic precision and reliability. Additionally, by mitigating the impact of class imbalance, SMOTE contributes to the model’s generalizability and robustness, ensuring consistent performance across diverse patient populations and clinical settings. Overall, the integration of SMOTE into the proposed model significantly enhances its predictive capabilities, ultimately empowering healthcare practitioners with a more effective tool for accurate heart disease diagnosis and prognosis.

**Figure 1:**
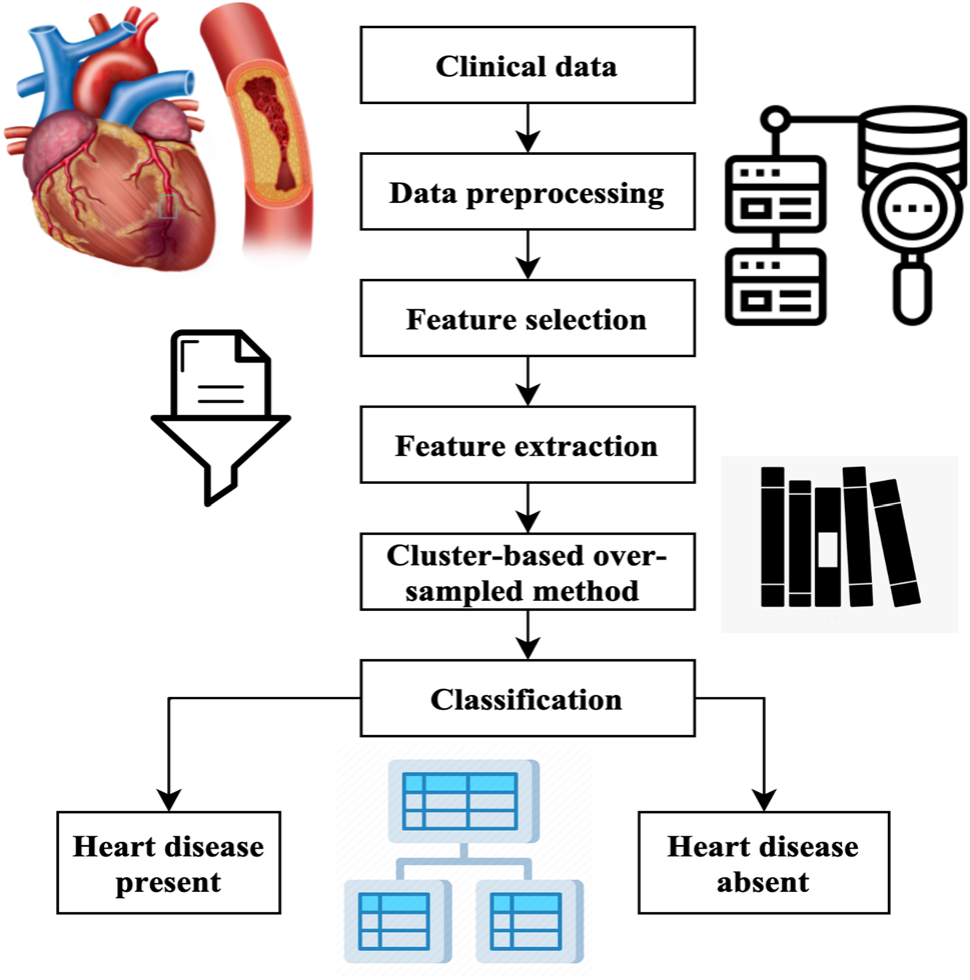
Workflow of the proposed method.

The methodology generates artificial instances of underrepresented categories, achieving a balanced distribution across both types. The process of classification involves the use of chosen characteristics and using various classifiers such as support vector machine (SVM)^21^, PCA^22^, linear discriminant analysis (LDA)^23^, naïve Bayes (NB)^24^, decision tree (DT)^25^, and random forest (RF)^26^. Ultimately, the classifier predicts an individual’s presence or absence of heart disease. The mechanism used in the suggested approach for heart disease forecasting is shown in Fig. 1.

### Pre-processing

This first phase is the primary step of the diagnostic procedure. The process consists of three stages: substituting absent qualities, eliminating duplications, and segregation. A characteristic’s missing value is substituted after a comprehensive examination of the patient’s age category, cholesterol levels, and blood pressure levels. If the majority of attribute values of a patient exhibit similarity, the corresponding value is replaced in the same spot. The redundancy reduction process aims to decrease the quantity of data by eliminating redundant or useless qualities. The patients are categorized according to the specific form of chest pain they exhibit, namely: (1) classic angina, (2) atypical angina, (3) non-anginal suffering, and (4) asymptomatic suffering.

### Feature selection

The GA is founded on the principles of natural selection. The algorithm produces many solutions within a single generation. Each solution is referred to as a genome. The aggregate of solutions within a single generation is often called the population. The algorithm undergoes several iterations across generations to provide an improved result. During each process iteration, genetic algorithms are used on genomes derived from previous generations to generate the generation. Selection, crossovers, and mutations are genetic operators often used in genetic algorithms.

**Figure 2:**
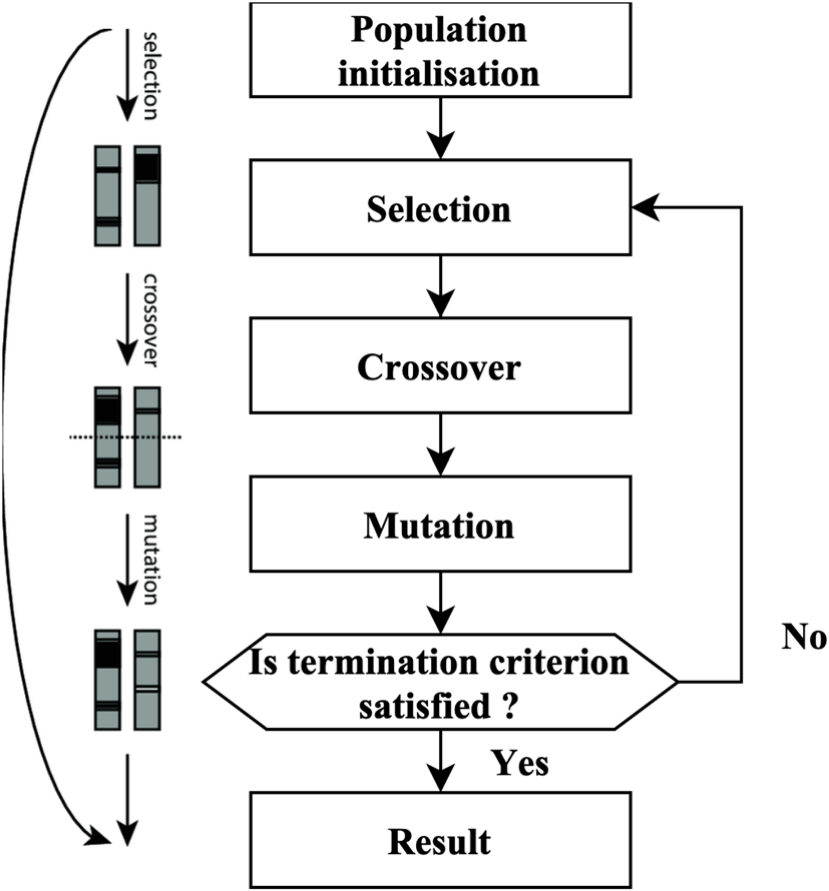
The process of GA.

The selection agent is responsible for choosing the most optimal people from every age group. A fitness function assesses each person’s fitness level about others within the general population. The genomes that a selection agent chooses are then put into the mating pool, contributing to the formation of the following generation. The crossover agent is a genetic algorithm technique that combines individuals from mating pools to generate improved offspring for generations. Various crossover agents exist, including single-point, two-point, and multipoint crossover. If a mixture is not introduced into the general population, individuals in the following generation will likely exhibit similarities to the preceding population. The variety is presented using a mutation manager, which introduces random alterations to the individuals. The process of the genetic algorithm’s development is shown in Fig. 2.

**Figure 3:**
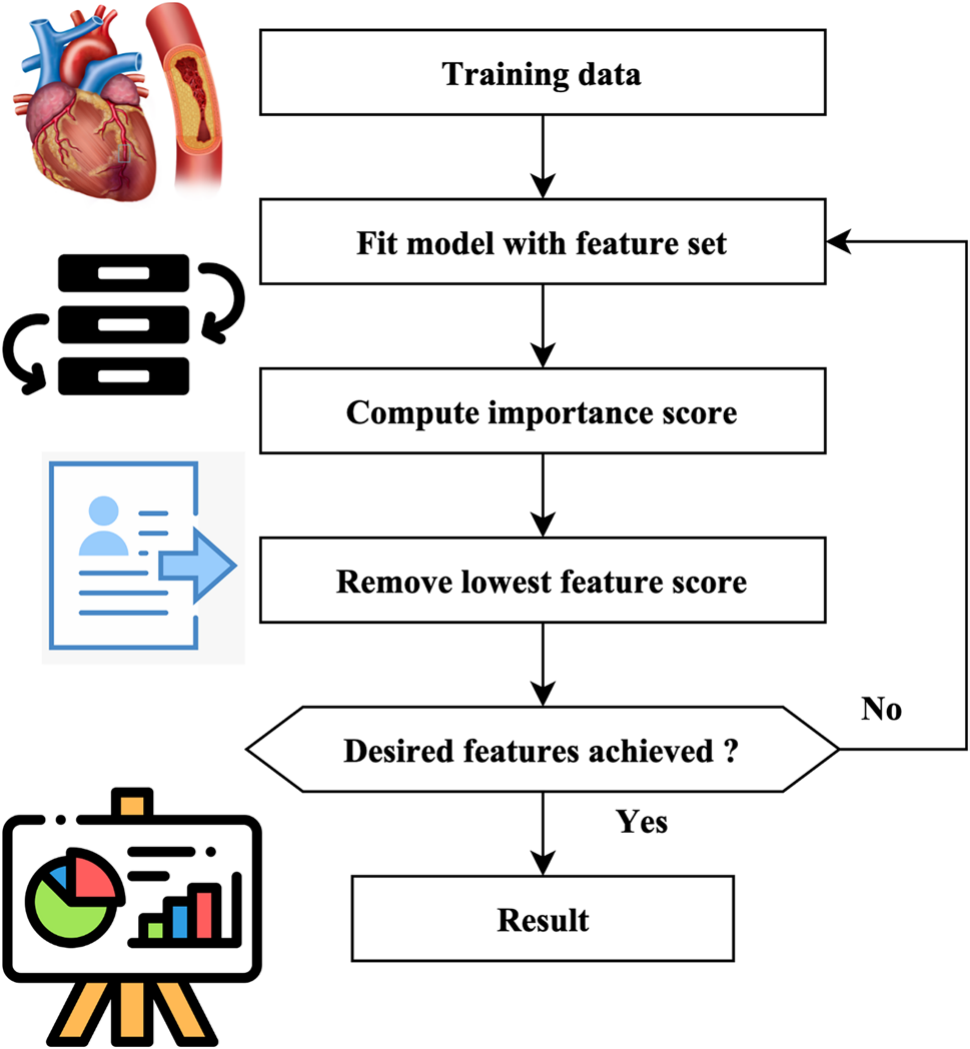
RFEM method workflow.

A total of 18 iterations have been used. A total of 80 genomes were chosen in every successive generation. A total of 40 optimal genomes were selected at each age and then transmitted to the next generation. A total of 40 genomes were picked randomly. A crossover rate of 5% and a mutation rate 0.05 were used. The user’s data is negated. The fitness of the genomes was determined by using the mean square error of the genome as the objective function. The RFEM method is an iterative process that recursively eliminates unnecessary characteristics. The classifier undergoes training using the provided training data, and afterward, the significance of the features is determined by calculation. The least influential elements are eliminated during each process iteration, and the model is retrained using the remaining subset of features. The procedures are executed iteratively until the necessary quantity of features is attained. The method receives the number of characteristics to be maintained as a parameter. The functionality of the RFEM method is shown in Fig. 3.

### Heart feature extraction

The investigation involves the extraction of diverse attributes from the medical data obtained via healthcare devices. The database comprises several physiological measurements, including heart rate, arterial pressure, and blood sugar level. To accurately determine the presence of cardiac disease, it is essential to extract critical characteristics, including statistical and temporal aspects. The equations provided below constitute the list of feature extraction.

The maximum voltage, total harmonic distortion (THD), heart rate, zero crossing rate (ZCR), entropy (ent), energy (eg), and standard deviation (SD) are expressed in Eqs. (1), (2), (3), (4), (5), (6), (7).

1 $${V}_{max}={\text{max}}\left\{V\right\}$$

2 $$THD=\frac{\sum_{i=0}^{n-1}{HC}_{i}}{{P}_{ff}}$$

3 $${H}_{r}=\frac{60}{{RR}_{t}}$$

4 $$ZCR=\frac{{S}_{c}}{{S}_{c}+{S}_{nc}}$$

5 $$Ent=\sum_{x=0}^{m-1}\sum_{y=0}^{n-1}\frac{{P}_{xy}}{{\text{log}}\left({P}_{xy}\right)}$$

6 $$Eg=\sum_{x=0}^{m-1}\sum_{y=0}^{n-1}{\left({P}_{xy}\right)}^{2}$$

7 $$SD=\frac{1}{M}\sqrt{\sum_{x=0}^{m-1}{\left({RR}_{x}-{RR}_{x-1}-k\right)}^{2}}$$

The voltage is denoted V, the harmonic component is denoted HC, the probability of frequency filter is denoted ${P}_{ff}$, the signal change and signal no change are expressed ${S}_{c} {\text{and}} {S}_{nc}$. The probability is expressed ${P}_{xy}$, and the deviation is denoted k. The collected data contains an interval referred to as RR. The variation is expressed in Eq. (8).

8 $$k=\frac{\sum_{x=0}^{m-1}{RR}_{x}-{RR}_{x-1}}{M-1}$$

The variable M represents the aggregate count of RR intervals in the database. The root mean square of the sum of the consecutive differences is expressed in Eq. (9).

9 $$R=\sqrt{\frac{\sum_{x=0}^{m-1}{RR}_{x}-{RR}_{x-1}}{M-1}}$$

${RR}_{x}$ refers to the integrated heartbeat, k represents the mean value, and ${P}_{xy}$ signifies the probability value. The qualities have been derived from the medical gadget data. The retrieved properties are retained as factors to forecast heart disease and alterations in the heart pattern. The ensuing section explains the intricate procedure involved in identifying heart disease.

### Cluster-based over-sampled method

A novel approach has been devised using resampling and grouping methodologies to handle unbalanced stroke data. Resampling techniques include both under sampling and oversampling methods. Under sampling is a technique to remove data points from the majority class specimens selectively. Oversampling is used to augment the representation of minority specimens. Grouping is the process of grouping data so that models within each group exhibit similar qualities.

**Figure 4:**
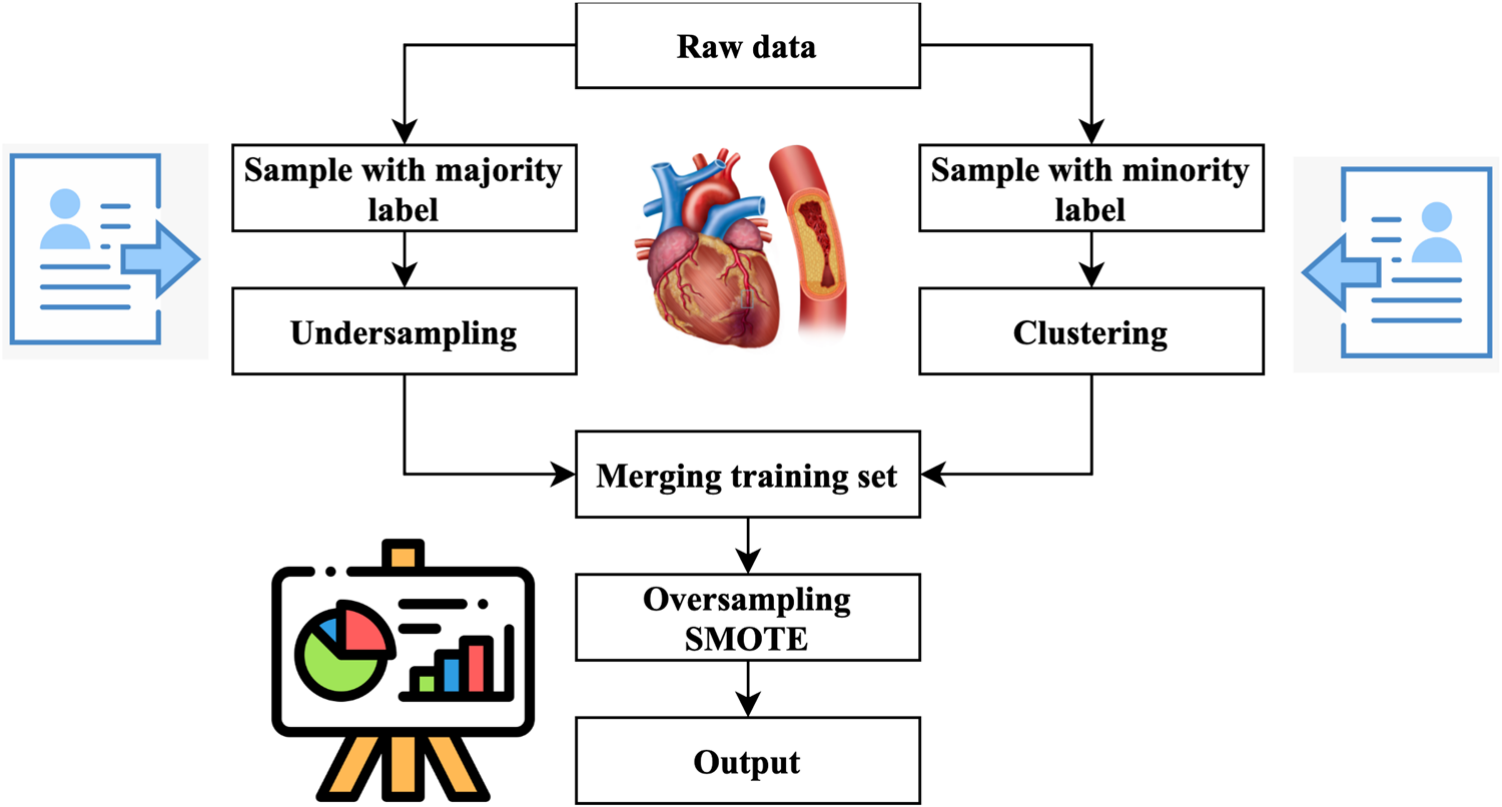
Cluster-based over-sampled method.

Figure 4 illustrates the primary operations inside the system that has been developed. The method performs an under sampling function on collecting specimens with majority labels. Clustering is performed on the subset of minority labels within the overall data set. The example set generated by the underestimating procedure is separated into distinct testing, training, and validation sets. This division is done according to the ratio of 8:1:1. The acquired training sets have been combined. The validation sets, and testing sets have been completed. Next, the SMOTE algorithm performs oversampling on the specimens with minority labels inside the merged training set, resulting in the acquisition of the final stage of training data^27^. In the concluding training set, the quantity of positive tests is about equivalent to the number of negative specimens, and each category of positive model is comprised. This training database offers an abundant number of examples that are used for the training of machine learning algorithms to extract characteristics. The method generates balanced activity, verification, and testing sets.

**Figure 5:**
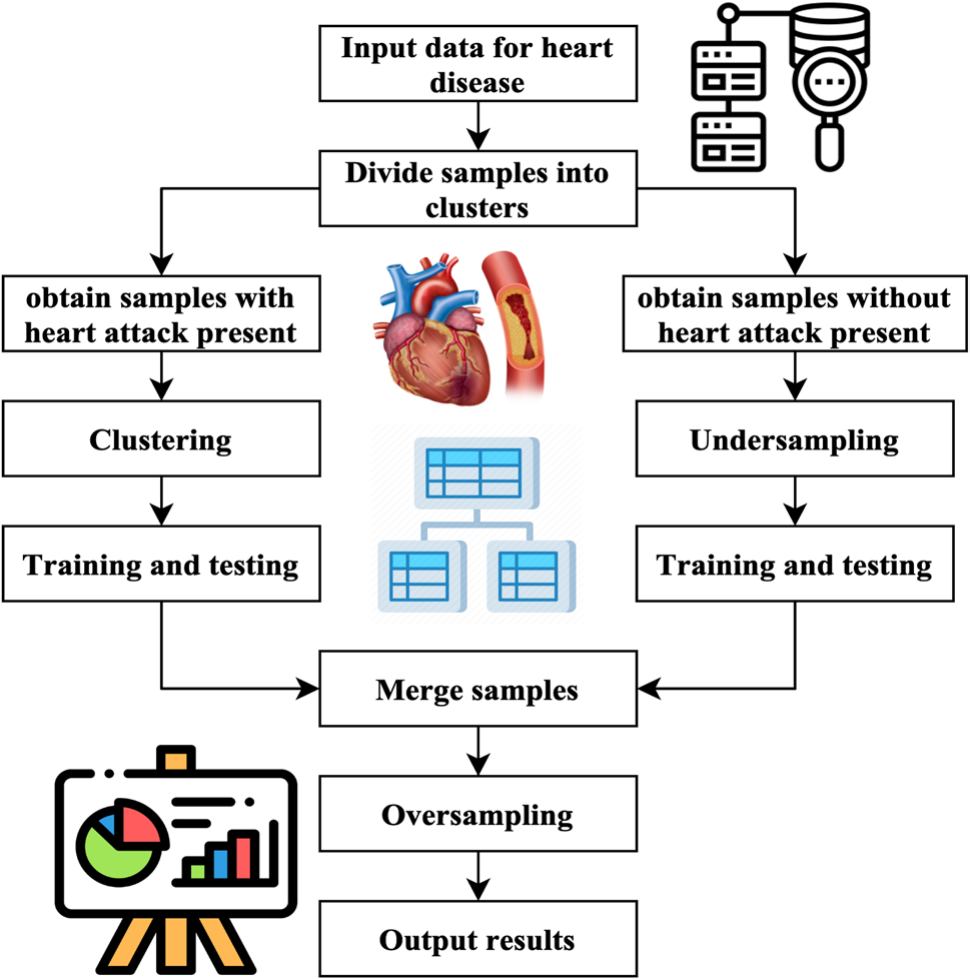
UCOM process for heart disease prediction.
  The research used a clustering algorithm to aggregate minority specimens, namely stroke specimens with cardiac arrest, into several clusters. Next, divide each group into three sets: the set for training, validation, and testing set. This division should be done by the ratio 8:1:1. The number of K is obtained by calculating the mean square error. The computation of the Sum of Squared Errors (SSE) is represented by Eq. (10).
  10 $$E=\sum_{i=0}^{{C}_{x}}{\left|i-{m}_{x}\right|}^{2}$$
  ${C}_{x}$ represents the x-th cluster, $i$ denotes the specimen inside ${C}_{x}$, and ${m}_{x}$ refers to the centroid of ${C}_{x}$
- (3) The process of dividing and combining clusters.
  The underspecified specimens and each cluster are divided into training, validation, and testing sets using an 8:1:1 ratio. These sets are combined to create a new activity, validation, and testing set. The three newly introduced groups consist of training, verification, and testing sets.
- (4) One approach to address the class imbalance in the training set is to use the SMOTE to specimen the pseudo training set.
  SMOTE method is a sampling technique designed to address imbalanced databases by generating synthetic specimens for the minority class. The method exhibits enhancements above the random sampling approach. The pseudo training set should be inputted into the SMOTE algorithm. The method employs a random selection process to choose specimens with a minority label and then identifies the closest neighbors. The system produces additional models with the minority label inside the set of centering specimens and their corresponding neighbour’s. The algorithm continues its execution until the count of samples with the minority label equals the count of models with the majority label. At this point, it produces the updated training set.

Figure 5 illustrates the UCOM process for heart disease prediction. The UCOM algorithm is comprised of three distinct parts. The number of individuals without heart attacks is much larger than those without, so the data set of individuals without heart attacks is underrepresented. The random underestimating procedure resulted in the selection of specimen number 120. The prediction outcome achieved with a specimen count of 120 demonstrates superior performance. A clustering operation is performed on the specimens associated with heart attacks to ensure the inclusion of distinct characteristics from each subgroup of close-distance data throughout the training process. To get a balanced distribution of specimens with and without heart attacks throughout the training, oversampling is used for the models with heart attacks.

- (1) The use of under sampling techniques to the majority class of negative specimens, namely those about stroke without heart attack cases. Most specimens, namely those without heart attack but with stroke, were picked randomly to create three subsets: a training subset, a validation subset, and a testing subset. The UCOM technique incorporates the parameter of randomized pick specimens, which depends on the data collection characteristics and the specimen set representing the minority label. The ratio of models with a heart attack to specimens without cardiac attack in the MIMIC-III database is 2:3, indicating an under sampling percentage^28^. In the MIMIC-III database, the stroke specimens database entails randomly selecting two-thirds of the stroke specimens, excluding those that include heart attacks.
- (2) Clustering the specimen set consisting of minority labels, namely stroke and heart attack data, is being performed.

### Classification method

The MLDCNN is a deep-learning neural network that utilizes the AEHOM for weight value optimization. Following the process of feature selection, the selected features are subjected to classification using the MLDCNN. The MLDCNN classifier receives each chosen component as its input in this context. The weights are arbitrarily given values associated with each piece of information. The concealed nodes inside the succeeding concealed layer compute the sum of the input value multiplied by the weight vector associated with all the input nodes connected to it. Random weight values have been shown to enhance the efficacy of the backpropagation process in obtaining the desired outcome. The optimization process is conducted in this manner. The activation procedure is then used, and the output of this layer is sent to the subsequent layer. The weights have a significant impact on the work of the classifier. The classification process in the MLDCNN involves a series of algorithmic phases, outlined as follows.

Step 1: Eqs. (11), (12) represent the feature values and their corresponding weights.

11 $${F}_{x}=\left\{{F}_{1},{F}_{2},\cdots ,{F}_{m}\right\}$$

12 $${W}_{x}=\left\{{W}_{1},{W}_{2},\cdots ,{W}_{m}\right\}$$

The symbol ${F}_{x}$ represents the input value, which represents a selection of n features denoted as ${F}_{1},{F}_{2},\cdots ,{F}_{m}$. Similarly, ${W}_{x}$ represents the weight value of ${F}_{x}$, which shows the appropriate weights for the n features as ${W}_{1},{W}_{2},\cdots ,{W}_{m}$.

Step 2: The inputs are multiplied by weight vectors that have been randomly selected, and the resulting products are then summed using Eq. (13).

13 $$M={F}_{1}{W}_{1}+{F}_{2}{W}_{2}+\cdots +{F}_{m}{W}_{m}$$

The input and the weight are denoted $F, and W$. $M$ represents the aggregate value.

Step 3: The objective is to ascertain that the activation function is expressed in Eq. (14) and the classification function is expressed in Eq. (15).

14 $${A}_{{f}_{x}}={C}_{x}\left\{\sum_{x=0}^{m-1}{F}_{x}{W}_{x}\right\}$$

15 $${C}_{x}={\text{exp}}\left(-{\left({F}_{x}\right)}^{2}\right)$$

The symbol ${A}_{{f}_{x}}$ represents the activation operation, whereas ${C}_{x}$ represents the exponential of ${F}_{x}$. The suggested system employs a Gaussian function as a kind of activation function.

Step 4: Assess the hidden layer output using a rigorous evaluation using Eq. (16).

16 $${Y}_{x}={B}_{x}+{C}_{1}{W}_{1}+{C}_{2}{W}_{2}+\cdots +{C}_{m}{W}_{m}$$

The bias value is denoted as ${B}_{x}$, whereas the weight across the hidden input and layers is specified as ${W}_{x}$. The classification function is denoted ${C}_{x}$.

Step 5: The three processes above are executed for every layer inside the MLDCNN. The output unit is assessed by aggregating the weights of every input signal to get the values of the neurons in the layer that produces signals expressed in Eq. (17).

17 $${R}_{x}={B}_{x}+{O}_{1}{W}_{1}+{O}_{2}{W}_{2}+\cdots +{O}_{3}{W}_{3}$$

The variable ${O}_{x}$ represents the value of the level that comes before the resultant layer, ${W}_{x}$ identifies the layer weights concealed, and ${R}_{x}$ denotes the measurement unit of the output.

Step 6: This stage involves the comparison of the output generated by the network with the desired target value. The disparity between these two quantities is often called the error signal. The mathematical expression for this disparity is expressed in Eq. (18).

18 $${E}_{x}={D}_{x}-{R}_{x}$$

The error indication, denoted as ${E}_{x}$, represents the discrepancy between the actual and desired output, specified by ${D}_{x}$. In this step, a comparison is made between the value of the output metric and the goal value. The associated mistake is identified. The error at the output is sent back to all other devices in the network by computing a value ${k}_{x}$ according to this mistake, denoted in Eq. (19).

19 $${k}_{x}={E}_{x}\left(f\left({R}_{x}\right)\right)$$

Heart rate is denoted ${R}_{x}$, and the error disparity is expressed ${E}_{x}$.

Step 7: The use of the backpropagation technique achieves the weight adjustment. The provided relation is presented in Eq. (20).

20 $${W}_{{c}_{x}}=\alpha {k}_{x}\left({F}_{x}\right)$$

The weight adjustment is denoted by ${W}_{{c}_{x}}$, the momentum is represented by $\alpha$, and ${k}_{x}$ refers to the mistake dispersed throughout the network. The weight values are adjusted using the AEHOM technique.

### AEHOM

The AEHOM framework is based on the notion that the elephant population is divided into several clans. Each clan has a distinct quantity of elephants. Typically, male elephants separate from their clans and choose a solitary lifestyle. The leadership of each clan is entrusted to the oldest female elephant, commonly called the matriarch. In a herd of elephants, the matriarchs are known to choose the most favorable option, whereas the male elephants’ attitude indicates the least favorable alternative. The elephant population is divided into several clans. Every individual, denoted as v, belonging to a particular family, indicated as c, is directed by the matriarch, denoted as ${E}_{n}$, who has the most significant fitness value within the given generation. The procedure above is represented by Eq. (21).

21 $${P}_{x+1,{E}_{n,v}}={P}_{{E}_{n,v}}+c\left({P}_{x,{E}_{n}}-{P}_{{E}_{n},v}\right){R}_{D}$$

${P}_{x+1,{E}_{n,v}}$ denotes the updated position of the variable u in the context of the algorithm c. ${P}_{x,{E}_{n}}$ reflects the prior location of u, while ${P}_{{E}_{n,v}}$ signifies the optimal solution of ${E}_{n,v}$. The variable c, which belongs to the interval [0, 1], determines the impact of the matriarch in the procedure. ${R}_{D}$ is a random number employed to enhance population diversity during the method's later phases. The optimal location of the elephant inside clan ${P}_{x+1,{E}_{n,v}}$ is revised via Eq. (22).

22 $${P}_{x+1,{E}_{n,v}}=l\left({P}_{c,{E}_{n}}\right)$$

The symbol l represents a parameter within the range of [0, 1]. This variable significantly determines how ${P}_{c,{E}_{n}}$ influences the overall outcome, as shown in Eq. (23).

23 $${P}_{c,{E}_{n}}=\frac{1}{{V}_{{E}_{n}}}\sum_{y=0}^{V{E}_{n}}{b}_{{E}_{n},y,l}$$

The variable ${b}_{{E}_{n},y,l}$ represents the yth dimension, where l is more than or equal to 1 and less than or equal to L. The variable L represents the total width of the space. $V{E}_{n}$ symbolizes the number of elephants in clan c.

Male elephants that separate from their social group are used to conduct exploratory activities. A subset of elephants exhibiting the lowest fitness values within each clan is assigned new locations, as Eq. (24) shows

24 $${P}_{w,{E}_{n}}={P\left({P}_{min}({\text{max}})k\right)}_{min}$$

The variables are defined as follows: ${P}_{min}$ represents the lower limit of the searching space. ${P}_{max}$ represents the upper bound of the searching area. The variable k is constrained to the interval [0, 1]. A stochastic variable generated from a uniform probability distribution.

After evaluating the placements of the elephants, mutation and crossing procedures are implemented to enhance the optimization process. The use of a two-point crossover is implemented. This approach involves the selection of two sites on the parental genome. The genetic material between these two specific locations undergoes reciprocal exchange between the parent’s genomes, resulting in the acquisition of the offspring’s genome. These points are evaluated in Eqs. (25) and (26).

25 $${x}_{1}=\frac{{P}_{x+1,{E}_{n}}}{3}$$

26 $${x}_{2}={x}_{1}+\frac{{P}_{x+1,{E}_{n}}}{2}$$

The mutation process (${P}_{x+1,{E}_{n}}$) entails exchanging a certain number of genes from each genome with novel genes. The exchanged genes refer to the genetically modified genes intentionally introduced into the genome without duplication. The procedure is iteratively executed until a solution exhibiting a higher fitness level is achieved.

The presented technique incorporates a comprehensive approach to predicting cardiac disease, which involves feature selection, data preparation, and machine learning algorithms. The data about patients is extracted and processed, resulting in the identification of valuable aspects. These characteristics are used in advanced machine learning methods, such as ensemble deep learning and classification methodologies. This merger aims to enhance precision and expedite the prognosis of cardiac disease with accuracy.

## Simulation analysis and findings

The performance of the suggested strategy is evaluated via thorough testing in the simulation part. Multiple metrics, including accuracy, sensitivity, specificity, and precision, are calculated to assess the predictive efficacy of the methodology. The effectiveness of the suggested strategy is validated by a comparative study conducted against current models.

### Databases

The UCI machine learning database, comprising four databases including the Cleveland database^29^, the Hungary database^30^, the Switzerland database^31^, and the Long Beach database, was utilized to gather data on heart illness^32^. Specifically, the Cleveland database was selected for this study due to its widespread use among machine learning researchers and its provision of comprehensive information. With a total of 303 entries, the Cleveland database originally contained 76 characteristics, although the dataset available in the repository includes information for only a subset of 14 features. Sourced primarily from the Cleveland Clinic Foundation, the Cleveland database serves as the primary data source for this study. The prediction of heart disease involves the consideration of 13 qualities, with one attribute serving as the output or anticipated attribute indicating the existence of heart disease in a patient.

Notably, the database from Cleveland includes a feature called “num,” which represents the finding of heart disease in individuals on a scale ranging from 0 to 4. A numerical value of 0 signifies the absence of heart illness, while values from 1 to 4 denote individuals afflicted with heart disease, with the scale indicating the degree of severity (with four being the most severe). Additionally, the study employed a modified bee method and k-fold cross-validation for robust analysis.

### Experimental setup for evaluation

The tests were conducted based on specific parameters and methodologies, including: (1) conducting the study using the entire database without employing any data-reducing methods, (2) experimenting with a reduced database by selecting a subset of the original data, and (3) experimenting with a modified bee method on the reduced database. The modified bee method, an adaptation of the traditional bee algorithm for optimization, was specifically tailored to enhance performance within the context of the study by iteratively exploring and optimizing parameters. These tests were executed on a system setup comprising an i5 CPU with 3 GB of RAM, utilizing the MATLAB 9.2 program for model construction. In each experiment, the data in the database was utilized to train and test the model, with different subsets or modifications applied as per the experimental conditions. Additionally, the study employed k-fold cross-validation, where the dataset was divided into k subsets, with each subset used as a testing set while the remaining data served as training sets. This process was repeated k times, and the performance metric computed the mean value over ‘k’ iterations to ensure robust evaluation of the model’s performance under varying conditions.

### Simulation results

The simulation results of the proposed method are analyzed, and the results are compared with the existing models.

**Figure 6:**
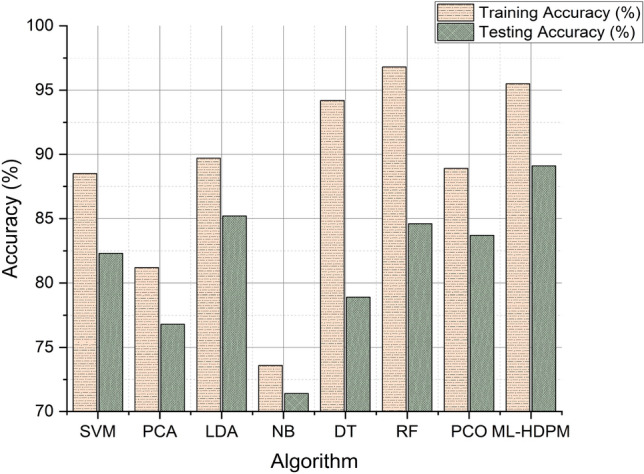
Accuracy analysis of predicting heart disease.

The accuracy analysis depicted in Fig. 6 showcases the effectiveness of the machine learning hybrid deep predictive model (ML-HDPM) approach in predicting heart disease. Compared to other algorithms, ML-HDPM achieves notably higher training accuracy (95.5%) and testing accuracy (89.1%). This significant improvement is attributed to the integration of various advanced methodologies within the model. Firstly, ML-HDPM employs sophisticated feature selection techniques to identify the most relevant attributes related to cardiovascular health, thereby enabling the model to discern subtle patterns indicative of heart disease. Additionally, the utilization of data balancing techniques ensures that the model is trained on a representative sample of both positive and negative instances, leading to more accurate predictions. Furthermore, the incorporation of deep learning capabilities through the MLDCNN architecture allows the model to automatically learn intricate patterns and relationships within the data, further enhancing its predictive accuracy. Lastly, optimization using the adaptive elephant herding optimization methodology (AEHOM) fine-tunes the model parameters and weights, resulting in improved accuracy. Overall, the synergistic integration of these methodologies enables ML-HDPM to accurately predict heart disease, thus demonstrating its potential to enhance clinical decision-making and patient care.

**Figure 7:**
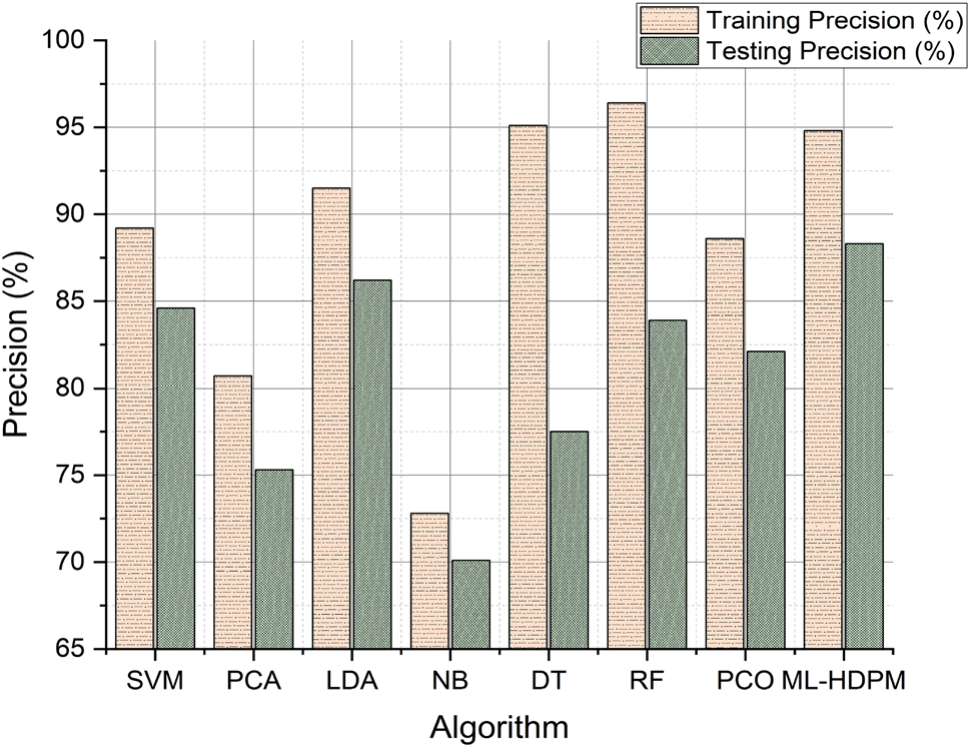
Precision analysis of predicting heart disease.

Figure 7 illustrates the precision analysis of heart disease prediction, showcasing the performance of the machine learning hybrid deep predictive model (ML-HDPM) technique. Notably, ML-HDPM achieves higher precision rates in both training (94.8%) and testing (88.3%) phases compared to alternative algorithms. This success can be attributed to the holistic approach adopted by ML-HDPM, which integrates several key methodologies. Firstly, the model employs advanced feature selection techniques to identify relevant attributes critical for accurate heart disease prediction. Additionally, ML-HDPM addresses data imbalances through effective data balancing methods, ensuring that the model is trained on a representative dataset. Moreover, the integration of deep learning capabilities using the MLDCNN architecture, enhanced by AEHOM optimization, further contributes to the model’s precision. By adeptly managing feature relevance and addressing data imbalances, ML-HDPM demonstrates enhanced accuracy in detecting heart disease patterns. These results underscore the importance of the proposed methodology in accurately diagnosing cardiac diseases, thus highlighting its significance in clinical decision-making and ultimately improving patient outcomes.

**Figure 8:**
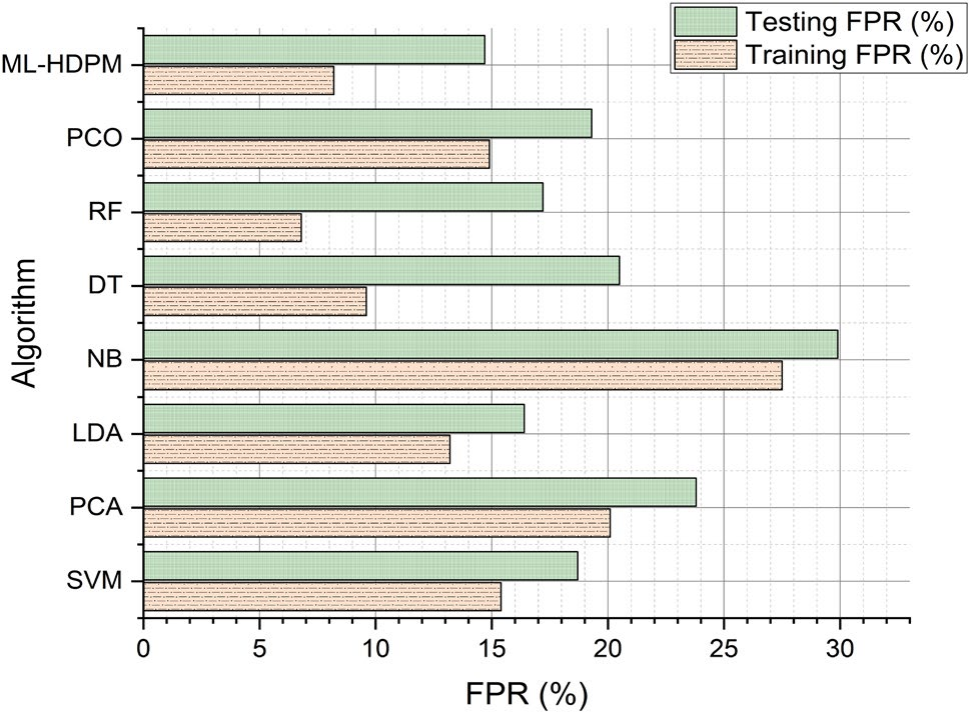
False positive rate analysis of predicting heart disease.

Figure 8 depicts the false positive rate (FPR) analysis of various methods for predicting heart disease, with the machine learning hybrid deep predictive model (ML-HDPM) technique exhibiting notably lower FPR values during both training (8.2%) and testing (14.7%) phases compared to alternative methods. This accomplishment can be attributed to the synergistic utilization of feature selection, data balancing, and enhanced deep learning components within the ML-HDPM framework. By employing these strategies, the model enhances its capability to accurately identify instances that do not have heart disease, thereby reducing the number of cases falsely identified as positive. Consequently, the suggested approach demonstrates efficacy in minimizing misclassification errors, a crucial aspect for achieving precise predictions of cardiac diseases and ultimately improving patient care outcomes.

**Figure 9:**
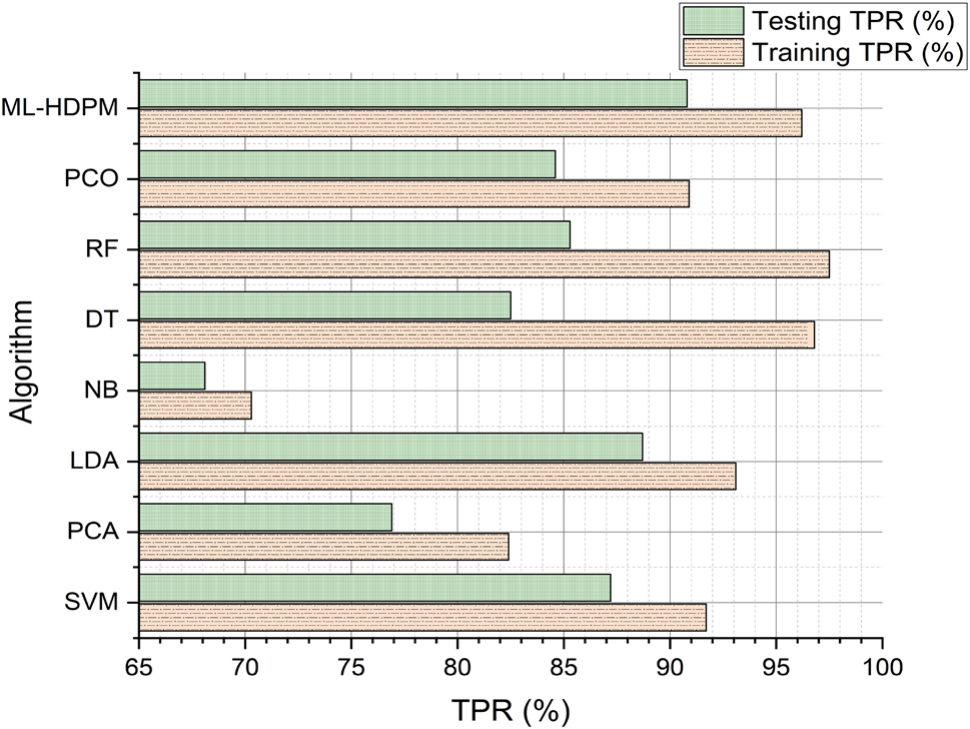
True positive rate analysis of predicting heart disease.

Figure 9 illustrates the true positive rate (TPR) analysis across various algorithms for predicting heart disease, highlighting the machine learning hybrid deep predictive model (ML-HDPM) technique’s superior performance. During both training and testing phases, ML-HDPM achieves notably higher TPR values, with 96.2% and 90.8%, respectively. This success can be attributed to the comprehensive methodology employed by ML-HDPM, which encompasses the identification of relevant features, data balancing, and optimization of deep learning techniques. By leveraging these strategies, ML-HDPM effectively utilizes its feature-rich and balanced database to accurately detect true positives, resulting in an enhanced TPR. These results underscore the potential of ML-HDPM in bolstering the prediction of heart disease, thereby facilitating more precise diagnoses and ultimately leading to better patient outcomes.

**Figure 10:**
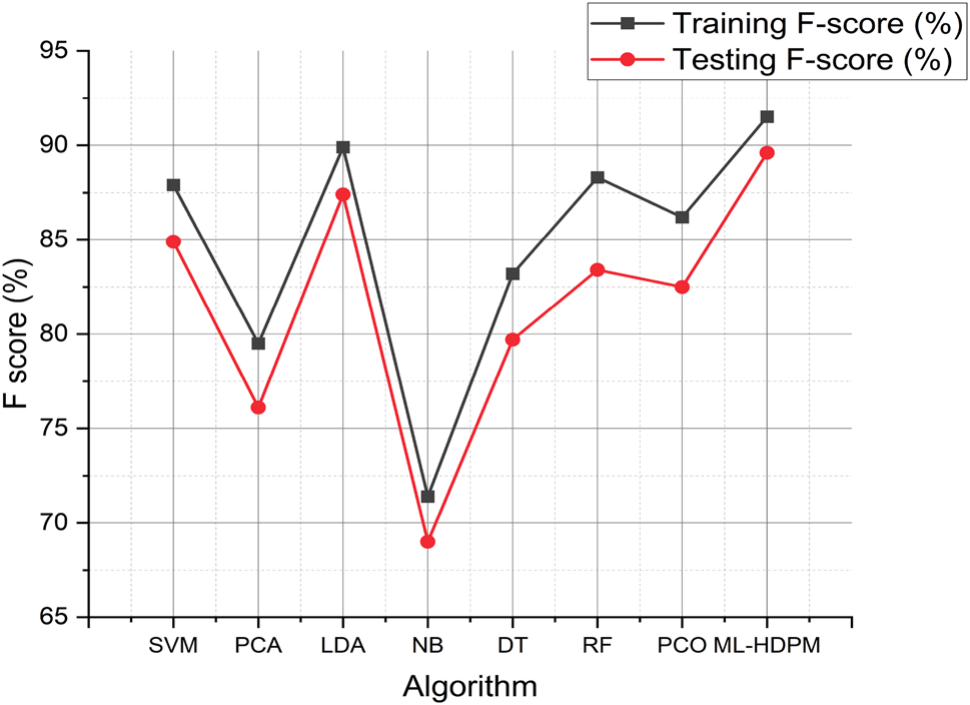
F score analysis of predicting heart disease.

The ML-HDPM technique has superior performance in terms of F-scores, achieving better scores in both training (91.5%) and testing (89.6%) compared to other algorithms. The F score results of the different models are expressed in Fig. 10. The success of this accomplishment is ascribed to the comprehensive nature of the methodology, which integrates feature selection, data balance, and deep learning optimization techniques. ML-HDPM successfully captures meaningful patterns in heart disease data, resulting in higher F-scores by establishing a balance between accuracy and recall. The results highlight the potential of ML-HDPM in accurately predicting heart disease, hence improving the whole diagnostic process and patient treatment.

**Figure 11:**
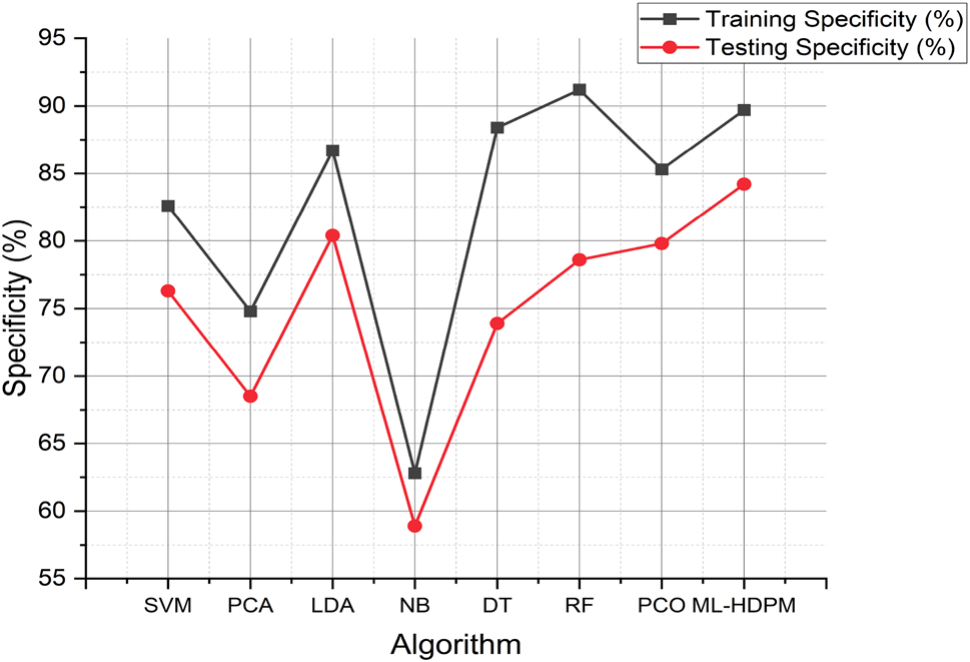
Specificity analysis of predicting heart disease.

The simulation findings presented in the study highlight the effectiveness of the machine learning hybrid deep predictive model (ML-HDPM) approach in accurately predicting heart disease. With a training accuracy of 95.5% and a testing accuracy of 89.1%, ML-HDPM outperforms other algorithms, demonstrating its exceptional performance in both sets. This superiority is further supported by the accuracy values of 94.8% for training and 88.3% for testing, underscoring the model’s effectiveness across different datasets expressed in Fig. 11. Moreover, ML-HDPM showcases a significant reduction in false positive rates (FPR), with rates of 8.2% during training and 14.7% during testing. This reduction in FPR is crucial as it indicates ML-HDPM’s ability to minimize misclassification errors, thereby improving the accuracy of heart disease prediction. Additionally, ML-HDPM exhibits higher true positive rates (TPR) compared to other algorithms, with rates of 96.2% during training and 90.8% during testing. These higher TPR values reflect ML-HDPM’s capability to accurately detect positive instances of heart disease, contributing to more reliable diagnostic outcomes.

Furthermore, ML-HDPM achieves balanced F-scores of 91.5% during training and 89.6% during testing, indicating a harmonious blend of precision and recall in its predictions. This balance is crucial for ensuring accurate and reliable heart disease prediction. Regarding specificity, ML-HDPM demonstrates superior performance, with specificity rates of 89.7% in training and 84.2% in testing, surpassing other algorithms. This notable difference in specificity can be attributed to the comprehensive methodology employed by ML-HDPM, which integrates feature selection, data balance, and deep learning optimization techniques. By effectively reducing false positive rates and properly recognizing real negatives, ML-HDPM enhances the precision of heart disease diagnoses, thereby improving patient care outcomes.

In summary, the considerable differences observed among the obtained results, particularly in terms of accuracy, false positive rates, true positive rates, F-scores, and specificity, highlight the superior performance of the ML-HDPM approach in predicting heart disease. The comprehensive methodology and integrated techniques utilized by ML-HDPM contribute to its effectiveness in improving accuracy, reducing misclassification errors, and enhancing diagnostic reliability, ultimately leading to better patient care outcomes.

## Conclusions

The research presents a comprehensive approach, ML-HDPM, aimed at enhancing the prediction of cardiovascular disease by addressing the limitations of traditional diagnostic methods. This approach combines various techniques such as feature selection, data balancing, and deep learning, utilizing the MLDCNN model improved with AEHOM.

The results of the study demonstrate promising performance metrics, with high training and testing accuracies, as well as precision rates. The approach also shows improvements in both false positive rates and true positive rates, indicating its potential for accurate prediction while minimizing false alarms. Additionally, the balanced F-scores suggest that the methodology maintains a good balance between precision and recall. These findings suggest that ML-HDPM has the potential to significantly improve the precision of diagnostic assessments for cardiovascular disease, thereby assisting healthcare professionals in making timely and accurate clinical decisions. However, the research also acknowledges the need for further exploration and refinement of the methodology, particularly regarding challenges related to data quality, feature selection, and optimization techniques. Future investigations should aim to expand the scope of inquiry to encompass a broader range of cardiovascular conditions and explore the practical implementation of the findings in real-world healthcare settings. Overall, the study underscores the transformative impact of advanced machine learning methodologies in improving the prediction and management of cardiovascular disease.

## Data Availability

The data presented in this study are available upon request from the corresponding author.

## Code availability

The custom code used in this study is openly available and can be accessed via https://github.com/prabukiwi1/Heart-Disease-Diagnosis. There are no restrictions to access the code, and researchers are encouraged to utilize it for further analysis or validation purposes.
